# An Exploration of the High Prevalence of Parkinson's Disease in a Rural Area of North West England

**DOI:** 10.1002/mdc3.70303

**Published:** 2025-08-25

**Authors:** Rosanna Varden, Ailish O'Callaghan, Richard Walker

**Affiliations:** ^1^ North Cumbria Integrated Care NHS Foundation Trust Carlisle UK; ^2^ Newcastle University Newcastle‐upon‐Tyne UK; ^3^ Northumbria Healthcare NHS Foundation Trust North Tyneside UK

**Keywords:** Parkinson's, prevalence, epidemiology, read code

## Abstract

**Background:**

Globally the prevalence of Idiopathic Parkinson's Disease (IPD) is rising. Increasing recognition is being given to the influence of environmental factors on this rise, although the precise impact of such factors remains poorly understood. We need accurate ways of measuring prevalence to understand regional and global trends.

**Objectives:**

To use case finding methodology to measure the crude, and age adjusted, prevalence of IPD in a rural area of North West England, and to describe the difference in results with those obtained using electronic record searching alone to understand the accuracy of such methods. To compare prevalence between subgroups to build hypotheses that could guide further research.

**Methods:**

Local Parkinson's service records from several sources were searched electronically and manually reviewed against validated diagnostic criteria. Figures were age adjusted according to the national denominator population.

**Results:**

The age adjusted prevalence of IPD was 201/100,000 (95% CI 186–217), higher than in any other published UK study using similar case finding methods. Numbers were higher using electronic diagnostic code searching alone, which may over estimate prevalence. No significant difference was seen between rural and urban areas, relative risk 0.92 (95% CI 0.80–1.06), although there was a trend to higher prevalence in historically industrial coastal areas.

**Conclusions:**

This study highlights some of the pitfalls in using large healthcare datasets to measure IPD prevalence. Overall, the prevalence of IPD is high in this predominantly rural area, and exhibits prevalence trends that warrant further investigation in relation to genetic and environmental factors.

The prevalence of Idiopathic Parkinson's disease (IPD) is rising globally.^1^ Increasing recognition is being given to the influence of environmental factors on this rise, although the precise impact of such factors remains poorly understood.^2^ We need accurate ways of measuring prevalence to understand regional and global trends. The cause of IPD is still not well understood, and the possible influence of environmental factors is being increasingly described.^3^ Within the United Kingdom (UK) however, it has been demonstrated that IPD prevalence and incidence has remained stable over recent decades.^4^, ^5^

North Cumbria, an area of North West England, is one of the most rural areas within the UK.^6^ Globally, concerns have been raised around rural living increasing IPD risk. This is likely to represent a complex interplay of environmental risk factors, but even when confounding factors are removed, studies have demonstrated an independent effect of rural living alone.^7^ Aside from its rural nature, North Cumbria has been home to a number of unique industries, including the Marchon manufacturing plant, copper and slate mining, and the Sellafield nuclear reprocessing site.^8^

Concerns have been raised around inequalities in managing chronic long term conditions, such as IPD, in rural and coastal areas. A UK report has highlighted gaps in understanding around health and social care provision for those living in such areas.^9^ This raises questions about the accuracy of understanding prevalence of disease in more remote areas. Accurate methods for measuring IPD prevalence on a large scale are needed. There is a move to using large electronic healthcare databases, which can facilitate measuring prevalence in large geographical areas, but whose accuracy is currently not fully understood. For example, discrepancies in diagnostic coding leading to an over‐estimation of disease, have been described in American veterans.^10^

This study aimed to use a case finding approach to measure the crude, and age adjusted, prevalence of IPD in this rural area, and compared this to results obtained using large database searching alone. It compared trends in prevalence across the region, and between subgroups, to give insight into possible environmental factors that may warrant further exploration.

## Methods

Health Research Authority (HRA) ethical approval was sought and issued on December 16, 2022. In the UK, Parkinson's care is provided by secondary and tertiary hospital services.

The study area was defined by CA1 to CA28 postcode as a continuous area of North Cumbria, which is made up of four districts or administrative areas of a population between 55,000 and 110,000 people. Within the study area, those living within a rural area were defined as those living within a settlement of less than 10,000 people. Prevalence rates were compared between districts and rural and urban areas to describe differences in trends.

The local movement disorder service is comprised of specialist clinicians employed by the local North Cumbria Integrated Care NHS Foundation Trust (NCIC). Some specialist care is provided by neighboring NHS trusts, which includes a tertiary neurology service provided by Newcastle Hospitals NHS Trust.

Details of cases were obtained from electronic lists of patients cared for by local IPD specialists, including doctors, specialist nurses and referral lists and historic neurology lists. All records are theoretically assigned a *Read Code* at the time of diagnosis, to include the terms Idiopathic Parkinson's, parkinsonism or a diagnosis of a confirmed parkinson's plus syndrome, based on diagnostic criteria. These diagnostic codes are added to an electronic record manually by clinicians or administrative staff as part of the referral process or diagnostic pathway. An electronic search was performed for those with these *Read Codes*, as well as all lists being hand searched for anyone with a diagnosis or symptoms suggestive of IPD such as tremor, bradykinesia.

Geriatricians and neurologists in NHS trusts of two neighboring counties were contacted and asked to provide lists of patients they cared for with IPD within the specified postcode area. These trusts used a combination of electronic record search from IPD specialists and outpatient Parkinson's lists. General practitioner (GP) practices within the area were asked to provide a list of patients on their records with a diagnosis of IPD, according to Read Codes, or on medications used to treat IPD which are rarely used in the treatment of other conditions. To ensure confidentiality, only a person's age, sex and first three letters of postcode were passed on. This allowed verification that the person resided within the study area. Lists were cross referenced with the list of those people already under the care of the NCIC PD service to prevent duplication. For those not known to this service, their GP contacted them on behalf of the study team to seek consent to pass on details to the researching team.

Inclusion criteria were those with a diagnosis of IPD, confirmed by the Movement Disorder Society Parkinson's Disease (MDS‐PD) diagnostic criteria.^11^ This meant the presence of supportive criteria and the absence of absolute exclusion criteria, authors recognize that red flags were accounted for as far as possible, however sometimes these take time to develop and may not have been present at the time of record review. Additional study inclusion criteria were residing in the study area and alive at the point of prevalence date, January 4, 2023. Regardless of assigned diagnostic *Read Code*, all identified clinical records were reviewed by the study team to confirm diagnosis. Those that had insufficient information on record initially were seen in the subsequent 15 months as part of routine clinical review to confirm diagnosis. Those who did not attend review by the NCIC service were contacted and offered a visit by the researcher to confirm diagnosis. Those who did not consent to this for whom there was insufficient information on record were not included.

As part of routine clinical review patients were asked where they had lived at birth and for the longest time during their working life, to help understand any potential influences of local environmental factors within the prevalent population.

Frequency of contact with specialist services for the prevalent population was described as contact with a specialist doctor or nurse within the preceding 6 or 12 months, to quantify any potential differences in service provision between rural and urban areas which may impact diagnosis and therefore prevalence rates.

Statistical analyses were conducted and crude prevalence estimates were described. Age adjusted prevalence estimates were calculated according to the UK population and weighted rates across age groups of 10 years. Figures pertaining to the UK denominator population were taken from the most recent 2021 census, published by the Office for National Statistics (ONS),^12^ where the denominator population comprised of 59.6 million people, and 318,000 people living in North Cumbria.

A 95% confidence interval (CI) was applied to each figure. Frequencies were expressed as % with 95% CI applied to percentages. Differences in subgroups where CIs did not overlap were deemed statistically significant. Relative risk (RR) was calculated to look at differences between male and female, and rural and urban populations.

## Results

**Figure 1:**
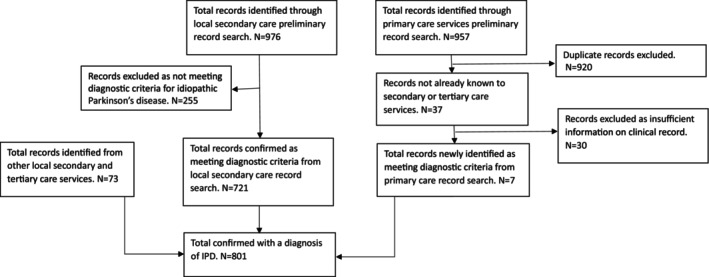
Flow chart indicating sources from where records where identified and how this informed the final record number.

Confirmed and excluded cases are summarized in Figure 1 (flow chart). On the point of prevalence date, January 4, 2023, 976 records were identified from the NCIC database search of elderly care and neurology records. Diagnoses at the time of first Read Code search are described in Table 1. The majority, 72% (95% CI 69.2–74.8), were confirmed as having IPD, although Table 1 highlights that the remaining 28% were wrongly identified by the electronic search, having symptoms more compatible with a different Parkinsonism or different diagnosis altogether.

**TABLE 1:** Actual diagnoses of identified records from preliminary search, confirmed following application of the MDS‐ PD criteria. Figures in () represent % of total

Diagnosis	Number identified
Idiopathic Parkinson's	703 (72.0)
Lewy Body dementia	25 (2.6)
Multisystem atrophy	10 (1.0)
Progressive supranuclear palsy	12 (1.2)
Corticobasal degeneration	2 (0.2)
Vascular Parkinsonism	75 (7.7)
Other	90 (9.2)
Insufficient information	59 (6.0)
Total	976

At initial secondary care record review, it was noted that Read Code search had wrongly included 90 individuals without IPD or Parkinsonism, when manually screened. These are described as “other” in Table 1 and diagnoses are described in Table 2. This represents 9.2% (95% CI 7.4–11.0) of the total NCIC identified population. Of these individuals, 29 had negative dopamine transporter (DAT) scans, which would rule out IPD in most cases. All these individuals wrongly identified were, or had been, on general neurology or outpatient Parkinson's lists. They had either been coded at the time of referral before review, or the code had not been changed if the diagnosis had been revised, for example in the case of essential tremor (ET). Of those diagnosed by a non‐Parkinson's specialist neurologist, nine were felt to be wrongly diagnosed with sufficient information held on record at time of first review, with the presence of red flags or absence of supportive criteria according to the MDS‐PD criteria.^11^

**TABLE 2:** Those captured by Read Code search without evidence of IPD or Parkinsonian syndrome. Figures in () represent % of total

No evidence of Parkinsonian syndrome	Number
Essential Tremor	45 (50.0)
Drug induced movement disorder	11 (12.2)
Musculoskeletal gait impairment	9 (10.0)
Other neurodegenerative disorder	11 (12.2)
Other	14 (15.6)
Total	90

At the time of initial NCIC secondary care record search, it was noted that for 59 individuals more information was needed before a definite diagnosis could be made. These are described above as having “insufficient information.” These records were reviewed again after 15 months and final diagnoses are described in Table 3. Of those confirmed as having IPD, eight were confirmed after DAT scan. The remaining 10 had confirmed diagnosis due to emerging clinical features. There was still diagnostic uncertainly for two individuals, who preferred a “watch and wait” approach rather than proceeding to DAT scan, these were not included in the final prevalence figure. Nine of these cases had negative DAT scans, ruling out IPD or a Parkinson's plus syndrome.

**TABLE 3:** Final diagnosis after review of records at 15 months, figures in () represent % of total

Review of diagnosis after 15 months for those with insufficient information at initial record search	Number
Idiopathic Parkinson's	18 (30.5)
Progressive supranuclear palsy	3 (5.1)
Other neurodegenerative disorder	3 (5.1)
Vascular Parkinsonism	15 (25.4)
Drug induced Parkinsonism	3 (5.1)
Essential tremor	9 (15.2)
Musculoskeletal gait impairment	6 (10.2)
Insufficient information	2 (3.4)
Total	59

A similar database search to that conducted for the NCIC records was conducted in Primary care. Although nothing is known about all these individuals it is interesting that this is a similar likely over estimate to the figure describe above from NCIC records. Of the 35 GP surgeries in North Cumbria contacted about participating further in the study, 17 surgeries (48.6%) agreed to participate. This included GP surgeries across the region, representing all four local authority areas equally.

A total of 801 individuals with IPD were identified as living in North Cumbria and alive at the point of prevalence date. The denominator population as recorded at the 2021 UK census for North Cumbria was 318,232. The crude prevalence of IPD was 252/100,000 (95% CI 234–269). The age‐adjusted prevalence was 201/100,000 (95% CI 186–217) using direct standardization to the UK population at the 2021 census.

The mean age was 74.7 years, 488 were Male and 313 were Female. The crude prevalence of IPD was statistically significantly higher for males than females, 310/100,000 (95% CI 283–338) and 194/100,000 (95% CI 173–216), respectively. The RR of having IPD for males compared to females in North Cumbria compared to the denominator population was 1.60 (95% CI 1.39–1.84). Of the prevalent population, 346 lived in an urban area and 455 lived in a rural area. The crude prevalence of IPD was not statistically different for rural and urban areas, at 243/100,000 (95% CI 221–266) and 264/100,000 (95% CI 236–291), respectively. There was no difference in average age between those living in rural and urban areas. The RR of having IPD living in rural, compared to urban areas in North Cumbria, compared to the denominator population was 0.92 (95% CI 0.80–1.06).

Crude and age adjusted prevalence for each district is shown in Table 4. Prevalence was highest in the district of Allerdale, a predominantly coastal and historically industrial area to the West of North Cumbria, and lowest in the district of Eden, a rural area of central North Cumbria where agricultural industries are the largest employer. However, the difference did not reach statistical significance.

**TABLE 4:** Crude and age adjusted Parkinson's prevalence by district in North Cumbria. Figures in () represent 95% confidence intervals

District	Number of cases	Denominator Population	Crude prevalence/100,000	Age adjusted prevalence/100,000
Allerdale	276	96,151	287 (253–321)	218 (189–248)
Carlilse	254	110,023	231 (203–259)	198 (172–224)
Copeland	137	57,323	239 (199–279)	196 (159–231)
Eden	134	54,735	245 (203–286)	177 (140–210)
North Cumbria Total	801	318,232	252 (234–269)	201 (186–217)

Of the individuals identified from NCIC records, historic postcode data were obtained for 541 (75.0%) out of the 721 individuals. Of these individuals 478, 88.4% (95% CI 85.7–91.1), had lived in North Cumbria for the longest duration of their working life, giving a crude prevalence of 150/100,000 (95% CI 137–164). This is an absolute minimum estimate of those potentially exposed to local environmental factors as it is recognized that there are missing data. However this conservative estimate is still higher than many other UK prevalence figures.^4^

Contact with specialist services for those cared for by the NCIC service, compared between rural and urban areas, are described in Table 5. There was no significant difference seen in contact between rural and urban areas.

**TABLE 5:** Numbers receiving formal service provision within the North Cumbria Parkinson's service, compared between rural and urban areas and areas of deprivation. Figures in () represent % of the total

Service provision	Total	Rural areas	Urban areas
No service contact last 12 months	50 (6.9)	25 (50.0)	25 (50.0)
Face to face Dr contact last 6 months	523 (72.5)	300 (57.4)	223 (42.3)
Face to face PDNS contact last 6 months	125 (17.3)	68 (54.4)	57 (45.6)
Phone PDNS contact last 6 months	71 (9.8)	35 (49.3)	36 (50.7)
Considered for advanced therapies	23 (3.2)	16 (69.6)	7 (30.4)
Total NCIC prevalent population	721	397	324

## Discussion

These results demonstrate a higher age adjusted prevalence of Parkinson's, 201/100,000, than any other UK study adopting similar case finding methodology, where age adjusted prevalence rates ranged from 105 to 168/100,000.^4^ The most recent of these studies was carried out in 2007, 16 years before this study. It is understood that globally Parkinson's prevalence is increasing^13^ although age will be a contributing factor, this should not affect age adjusted rates. Conversely studies show no changes suggesting rising rates in the UK,^4^ and the use of large healthcare datasets has shown no overall trend to rising incidence.^5^ Both globally and in the UK it is important to recognize that prevalence rates will be influenced not only by incidence, but also mortality and disease duration, as well as access to healthcare and timely diagnosis.

This study has the second biggest denominator population compared to other published UK studies^4^ which is likely to have improved accuracy and is reflected in the relatively narrow CI of the overall age adjusted prevalence figure.

We note that inclusion criteria were strict, including only those for whom there was sufficient information on record to confirm diagnosis against MDS‐PD criteria. We note however the proportion of wrongly identified records identified using Read Code search alone. Read Code searching identified 976 and 957 records from secondary care and primary care retrospectively. This is higher than the final 801 confirmed cases. IPD is predominantly a clinical diagnosis, made by a specialist, in conjunction with DAT scanning and evolving clinical features. This distinguishes IPD from other conditions exhibiting Parkinsonian features. This means that diagnosis is often not made at time of first review or is revised over time. These results have demonstrated this can result in Read Code discrepancy. The most commonly included misdiagnosis was ET. A study of American veterans has also highlighted significant record coding inaccuracy, with accuracy being as low as 46.5%, and also described greater discrepancy in minority groups.^10^ This study also described inclusion of those with ET, as well as drug induced parkinsonism.^10^

Numbers described with vascular Parkinsonism and conditions such as DLB are likely to be an underestimate and these are not always routinely cared for by secondary care Parkinson's services. It is also recognized that sometimes symptoms associated with Parkinsonism take time to develop and may not have been apparent at the time of record review.

We recognize that our methodology was very labor intensive. Many studies are moving to utilizing large healthcare datasets to capture larger volumes of data. Improved understanding of the pitfalls of such methods is likely to improve accuracy over time. A study using deep learning algorithms to analyze not only current symptoms but potential prodromal features demonstrated a potential cost effective way of measuring prevalence patterns.^14^ Such algorithms could be developed to help exclude common symptom patterns in conditions such as ET, DLB or conditions leading to vascular Parkinsonism.

In this study proportionally more men were affected than in other UK studies, RR 1.6 times higher in men than women. In Northumberland 52.8% of participants were male.^15^ In West Scotland crude male IPD prevalence was 133.1/100,00 and crude female prevalence was 105.3/100,000.^16^ Global studies have described RR being 1.5 times greater in men than women.^17^ Reasons for the higher proportion of men affected are unclear. It is unlikely that there is more health seeking behavior amongst the male population in this area, compared to other areas. It does raise the possibility of the influence of environmental occupation exposures, explored below, with men historically working in farming and heavy industry in the area.^18^

There are variations in age adjusted prevalence within the region, although numbers overall in each area are small, CIs are wide, and differences did not reach statistical significance. Allerdale, a rural coastal region with a history of heavy industry, demonstrated the highest age adjusted prevalence while Eden, a rural inland area, demonstrated the lowest prevalence. Overall within North Cumbria, these data have not demonstrated higher prevalence in rural compared to urban areas, although this is a predominantly rural area overall. This would suggest that rural living alone does not account for prevalence differences.

In the UK, studies carried out in the North of England, with identical methodology, demonstrated no higher prevalence in rural Northumberland^15^ compared to the more urban area of North Tyneside.^19^ Conversely rural living in Scotland has been associated with higher IPD prevalence.^16^ Attempts to quantify the effects that rural living may have on influencing prevalence have been made^20^, ^21^ but the precise relationship remains unclear. It has been hypothesized that those living rurally having poorer infrastructure and access to healthcare.^22^ Although rates may be higher in these areas, they are not being diagnosed, or are diagnosed later. These study data would not support this as those in rural areas were demonstrated to receive similar specialist contact to those in urban areas.

The environmental factors linked to rural living have previously been described as increasing Parkinson's prevalence. This includes pesticides, first described in 1987,^23^ and more recently in a number of studies highlighting the effect of paraquat and rotenone.^24^, ^25^

Other chemicals and heavy metals related to industry have been attributed to causing IPD including trichloroethylene (TCE)^26^ and lead.^27^ Industrial impacts relevant to this population include sulfuric acid production, iron and copper mining. Risks relating to these industries and IPD have not previously been well explored. Previous research has attempted to look at the local environmental impact of the Sellafield nuclear reprocessing site^8^ but the impact of radio nuclear isotopes is poorly understood.

Most individuals in the study have lived in Cumbria all their life, and likely share genetic similarities as well as similar environmental exposures. More research is needed in understanding the prevalence of genetic factors associated with IPD causation in this area.

Having a large denominator population increased sample size and is likely to have improved statistical power. This study utilized case finding methodology so would not have captured those not yet diagnosed and inclusion criteria overall were strict with some potential cases from primary care not analyzed. This age adjusted figure therefore could be an underestimate. Although more records were identified through wider Read Code searching, this study has demonstrated incorrect diagnosis in many of these cases.

Other UK studies have also raised concern that rural areas lacking in healthcare infrastructure are more likely to underestimate IPD prevalence,^28^ although a strength of this study is the ability to demonstrate similar service contact between rural and urban areas.

Knowing that most of the prevalent population have lived and worked in North Cumbria for most of their life is a strength here, which may help the design of future epidemiological studies to look at the influence of environmental and genetic factors on Parkinson's prevalence.

In conclusion, this comparatively large UK prevalence study conducted in a rural area of North West England has demonstrated a higher age adjusted prevalence of IPD than any other UK study conducted using similar methods. A trend to higher prevalence in historically industrial rural coastal areas has been demonstrated. North Cumbria has some unique environmental factors that would warrant further exploration in relation to IPD and the interplay between its potential genetic influences. Robust evidence could be obtained from epidemiological studies such as case control or retrospective cohort studies. Whereas it is recognized that this methodology was labor intensive, it has highlighted some discrepancies in figures that would have been obtained using electronic record Read Code searching alone. Insights into this may help improve such search strategies, improving the accuracy of future prevalence figures.

## Author Roles

(1) Research project: A. Conception, B. Organization, C. Methodology, D. Investigation, E. Supervision. (2) Statistical Analysis: A. Data Curation, B. Format Analysis. (3) Manuscript Preparation: A. Writing of the first draft, B. Review and Critique.

R.V.: 1A, 1B, 1C, 1D, 2A, 2B, 3A.

R.W., A.O.: 1A, 1C, 1E, 3B.

## Disclosure

**Ethical Compliance Statement:** United Kingdom Health Research Authority (HRA) ethical approval was sought and issued on December 16, 2022, following review from the Brighton and Sussex Research Ethics Committee, ref 22/LO/0749, IRAS 315626. All patient identifiable data were anonymized according to HRA confidentiality advisory group guidelines. Informed patient consent was not necessary for this work. We confirm that we have read the Journal's position on issues involved in ethical publication and affirm that this work is consistent with those guidelines.

**Funding Sources and Conflict of Interest:** No specific funding was received for this work. The authors declare that there are no conflicts of interest relevant to this work.

**Financial Disclosures for the Previous 12 Months:** RV's role is funded by the UK Charity Parkinson's UK, employed by North Cumbria Integrated Care NHS Foundation Trust and affiliated to Newcastle University. She is in receipt of a grant from Parkinson's UK to set up a project to provide more equitable Parkinson's care in the local service, £8500 18 months from September 2023, and a grant from the British Geriatrics Society Movement Disorder Special Interest Group, £1931.98 August 2022. The funders had no role in the design of the research, results or contents of this paper. RW is a Consultant Physician and Honorary Professor of Aging and International Health, North Tyneside General Hospital and Population Health Sciences Institute, Newcastle University. Chief investigator for current grant funding: Michael J. Fox Foundation (MJFF, GP2). Aligning Science across Parkinson's initiative, the genetic profile of Parkinson's Disease in Africa, 800,000 USD October 2023 for 2 years. NIHR Global Health Research Group on Transforming Parkinson's Care in Africa (TraPCAf), Research costs £2,999,500.00, 4 years from September 1, 2022. These funders had no role in the design of the research, results or contents of this paper. AO'C is a Consultant Physician employed by North Cumbria Integrated Care NHS Foundation Trust and affiliated to Newcastle University, with no grants to declare. The authors have no other conflicts of interest to declare.

## Data Availability

The data that support the findings of this study are available from the corresponding author upon reasonable request.
